# Scandium-44 Radiolabeled Peptide and Peptidomimetic Conjugates Targeting Neuropilin-1 Co-Receptor as Potential Tools for Cancer Diagnosis and Anti-Angiogenic Therapy

**DOI:** 10.3390/biomedicines11020564

**Published:** 2023-02-15

**Authors:** Katarzyna Masłowska, Patrycja Redkiewicz, Paweł Krzysztof Halik, Ewa Witkowska, Dagmara Tymecka, Rafał Walczak, Jarosław Choiński, Aleksandra Misicka, Ewa Gniazdowska

**Affiliations:** 1 Centre of Radiochemistry and Nuclear Chemistry, Institute of Nuclear Chemistry and Technology, 03-195 Warsaw, Poland; 2 Department of Neuropeptides, Mossakowski Medical Research Institute Polish Academy of Sciences, 02-106 Warsaw, Poland; 3 Faculty of Chemistry, University of Warsaw, 02-093 Warsaw, Poland; 4 Heavy Ion Laboratory, University of Warsaw, 02-093 Warsaw, Poland

**Keywords:** angiogenesis, theranostic ^43,44,47^Sc-radiopharmaceuticals, anti-angiogenic therapy, neuropilin-1, VEGF-A_165_/NRP-1 complex inhibitor, angiogenic factors, A7R peptide, retro-inverso isomer ^D^R7A, peptidomimetics

## Abstract

Pathological angiogenesis, resulting from an imbalance between anti- and pro-angiogenic factors, plays a pivotal role in tumor growth, development and metastasis. The inhibition of the angiogenesis process by the VEGF/VEGFR-2/NRP-1 pathway raises interest in the search for such interaction inhibitors for the purpose of the early diagnosis and treatment of angiogenesis-dependent diseases. In this work we designed and tested peptide-based radiocompounds that selectively bind to the neuropilin-1 co-receptor and prevent the formation of the pro-angiogenic VEGF-A_165_/NRP-1 complex. Three biomolecules, A7R and retro-inverso ^D^R7A peptides, and the branched peptidomimetic Lys(hArg)-Dab-Pro-Arg (K4R), conjugated with macrocyclic chelator through two linkers’ types, were labeled with theranostic scandium-44 radionuclide, and studied in vitro as potential targeted radiopharmaceuticals. ELISA (enzyme-linked immunosorbent assay) studies showed no negative effect of the introduced biomolecules’ changes and high NRP-1 affinity in the case of A7R- and K4R-radiocompounds and a lack affinity for ^D^R7A-radiocompounds. All radiopeptides showed a hydrophilic nature as well as high stability against ligand exchange reactions in cysteine/histidine solutions. Unfortunately, all radiocompounds showed unsatisfactory nano-scale stability in human serum, especially for use as therapeutic radioagents. Further work is ongoing and focused on the search for angiogenesis inhibitors that are more human serum stable.

## 1. Introduction

Angiogenesis is the process of formation of a network of blood vessels that occurs mainly in embryonic development, but also during wound healing and in the development of neoplasms, especially in their early stages [1,2,3]. Work on angiogenesis was initiated in the second half of the 20th century by Judah Folkman ([4] and papers cited therein, [5,6]) and showed that cancer cells need their own blood vessels to survive and grow. Above the critical number of cancer cells (on the order of 10^6^, corresponding to a tumor diameter of about 1–3 mm), simple diffusion is no longer sufficient for the tumor to receive enough oxygen and nutrients for further growth and metastasis [5,6]. Since tumor growth is angiogenesis dependent, the inhibition of angiogenesis may be a therapeutic target in the treatment of cancer (anti-angiogenic therapy, AAT) [5,7,8,9]. One of the main stages of the angiogenesis process is the interaction between the pro-angiogenic vascular endothelial growth factor (VEGF-A_165_), its receptor (VEGFR-2), and the neuropilin-1 (NRP-1) co-receptor [10,11,12,13,14,15], resulting in formation of ternary VEGF-A_165_/VEGFR-2/NRP-1 complex. Due to NRP-1 overexpression on many types of cancer cells ([16] and papers cited therein, [17]) as well as the relatively well-known mechanisms of the formation and action of the VEGF-A_165_/NRP-1 complex, the design of novel compounds that can block the formation of this complex is a promising direction in the search for anti-angiogenic and anti-cancer drugs [12,14,18,19]. Such inhibitors of VEGF-A_165_/NRP-1 complex formation may be compounds with high affinity for the NRP-1 co-receptor. These inhibitors labeled with diagnostic (emitters of gamma or beta plus radiation) or therapeutic (emitters of Auger electrons and alpha or beta minus radiation) radionuclides may find application as potential diagnostic or therapeutic receptor radiopharmaceuticals in targeted radionuclide therapy (TRNT) [8,20,21,22].

Among the radionuclides used in nuclear medicine, three isotopes of scandium (^43^Sc, ^44^Sc and ^47^Sc) deserve special attention [23,24,25,26,27]. Isotopes ^43^Sc (t_1/2_ 3.89 h, β^+^ E_max_: 88%, 1.20 MeV) and ^44^Sc (t_1/2_ 3.97 h, β^+^ E_max_: 94%, 1.47 MeV) are beta plus (β^+^) radiation emitters and may be used in diagnostic radiopharmaceuticals for positron emission tomography (PET) imaging, while the beta minus (β^−)^ radiation emitter ^47^Sc (t_1/2_ 3.34 d, β^−^ E_max_: 68%, 600.10 keV and 440.72 keV) may be used in radiopharmaceuticals dedicated to therapy. These isotopes are an example of theranostic radionuclides, and the radiopreparations containing these radionuclides are theranostic radiopharmaceuticals with identical or very similar physicochemical and biological properties. Both diagnostic and therapeutic radiopharmaceuticals have the same receptor affinity and pharmacokinetics in the patient’s body. Effective molecular imaging and disease diagnosis with the use of such a theranostic radiopharmaceutical pair (differing only in the type of emitted radiation) will allow for maximum therapeutic effect. The application of a theranostic radiopharmaceutical pair is the basis of the personalized therapy that is more and more often used in nuclear medicine.

Studies of the crystallographic structure of the VEGF-A_165_/NRP-1 complex showed that NRP-1 binds to VEGF-A_165_ through the b1 and b2 subdomains of neuropilin, as a result of which the binding of VEGF-A_165_ to VEGFR-2 is enhanced [18,28,29,30,31]. These studies also showed that the C-terminal arginine residue of VEGF-A_165_, which fits perfectly into the binding pocket in the NRP-1 b1 subdomain, plays an essential role in this interaction [14,18,31,32]. Adding another amino acid, and even blocking the free carboxyl group of this arginine residue (e.g., by amidation), eliminated VEGF-A_165_ activity [18,33]. The discovery that other ligands capable of binding to NRP-1 also had such a C-terminal arginine or lysine fragment led to the concept of the existence of C-End-Rule (CendR), according to which peptides or peptidomimetics with C-terminal arginine (or rarely lysine) residue (generally containing the R/KXXR/K amino acid sequence) may efficiently bind to the binding pocket of the NRP-1 b1 subdomain [12,14,18,32,33].

There are many works in the literature on substances with peptides, peptidomimetics or simple organic molecules’ structure, targeting NRP-1 ([18,34] and papers cited therein). One of them is the Ala-Thr-Trp-Leu-Pro-Pro-Arg (A7R) heptapeptide, found by a mutated phage library screening, that selectively inhibits the binding of VEGF-A_165_ to NRP-1, reduces angiogenesis and breast cancer growth in vivo [35,36,37]. Based on the structure of A7R and our previous study [34], the branched peptidomimetic Lys(hArg)-Dab-Pro-Arg (K4R) was designed, which according to the ELISA (enzyme-linked immunosorbent assay) test is a stronger inhibitor of VEGF-A_165_ binding to NRP-1, and at the same time, is more stable in human serum compared to A7R in micro-scale research [38]. Continuing the search for inhibitors of the VEGF-A_165_/NRP-1 complex that are stable in human serum at the nano-scale, we found information in the literature that the retro-inverso isomer of A7R (dArg-dPro-dPro-dLeu-dTrp-dThr-dAla; termed here ^D^R7A) is characterized by both an affinity for NRP-1 and a better serum stability [39,40]. This retro-inverso A7R modification involves both inversions of amino acid stereochemistry (replacement of L-amino acids with D-amino acids) and the reversal of peptide bonds.

The aim of the present work was to modify (attachment linkers and chelators) selected NRP-1 targeted biomolecules (A7R and ^D^R7A peptides and K4R peptidomimetic), to label them with radionuclide scandium-44, to investigate in nano-scale the physicochemical properties of the obtained radioconjugates from the point of view of their use as a theranostic radiopharmaceutical pair as well as to determine their affinity (using appropriate reference compounds containing stable isotopes) for NRP-1 tested with the ELISA assay and to check the effect of biomolecule modification on the parent biomolecule properties. Receptor radiopharmaceuticals based on optimally modified peptide VEGF-A_165_/NRP-1 complex inhibitors will be able to bind in a specific and selective manner with high affinity for a selected molecular target—the NRP-1 co-receptor. In such targeted anti-angiogenic radionuclide treatment, diagnostic and therapeutic radiocompounds designed in this work would enable both the real-time localization of cancer cells at the molecular level at a very early stage of tumor development by imaging the pathological VEGF-A_165_/NRP-1-related vascular system in its close microenvironment, as well as the selective inhibition of the formation of pathological vascularization and the destruction of previously located tumor cells. The combination of such targeted imaging and therapy in the concept of theranostic radiopharmaceuticals is a significant contribution to personalized nuclear medicine, increasing the effectiveness of treatment while maintaining an appropriate safety profile. In addition to radioconjugate syntheses using the theranostic isotope ^44^Sc, we also synthesized and tested the ^68^Ga- and ^177^Lu-^D^R7A radioconjugates, which allowed them to be compared with the corresponding radioconjugates based on A7R peptide ([^68^Ga]Ga-DOTA-Ahx-A7R, (**^68^Ga-1**), and [^177^Lu]Lu-DOTA-Ahx-A7R, (**^177^Lu-1**)), and K4R branched peptidomimetic ([^68^Ga]Lys(hArg)-Dab(Ahx-DOTA-Ga)-Pro-Arg, (**^68^Ga-2**), and [^177^Lu]Lys(hArg)-Dab(Ahx-DOTA-Lu)-Pro-Arg, (**^177^Lu-2**)) described in our previous publication [41].

## 2. Materials and Methods

### 2.1. Materials

Fmoc-Arg(Pbf)-Wang resin was obtained from Activotec (Cambridge, UK). Amino acids and coupling reagents were purchased from Iris Biotech (Marktredwitz, Germany).

Conjugates **1** (DOTA-Ahx-A7R, synthesis yield 37%, purity >98%) and **2** (Lys(hArg)-Dab(Ahx-DOTA)-Pro-Arg; DOTA-Ahx-K4R, synthesis yield 27%, purity > 95%), both contained 6-aminohexanoic acid (Ahx) as a linker were prepared manually by Solid Phase Peptide Synthesis (SPPS) method and standard Fmoc methodology according to the procedure described in detail in our previous publication [41] and in brief in Section 2.2.2. Syntheses.

Conjugate of A7R peptide coupled with DOTA chelator via Lys amino acid as a linker (conjugate **1bis**, Lys(DOTA)-A7R, purity > 99%) was purchased from CASLO ApS (Kongens Lyngby, Denmark), as a compound Lys(DOTA)-Ala-Thr-Trp-Leu-Pro-Pro-Arg in the form of lyophilized trifluoroacetate salt ready for further syntheses.

Conjugate of retro-inverso isomer of A7R peptide with DLys as a linker and DOTA chelator (conjugate **3**, ^D^R7A-^D^Lys(DOTA), purity > 99%) was purchased also from CASLO ApS (Kongens Lyngby, Denmark), as a compound dArg-dPro-dPro-dLeu-dTrp-dThr-dAla-dLys(DOTA) in the form of lyophilized trifluoroacetate salt ready for further syntheses.

Pooled 100% human serum (HS) was obtained from Innovative Research (Novi, MI, USA).

Recombinant human NRP-1 was purchased from BioLegend (San Diego, CA, USA), biotinylated VEGF-A_165_ (bt-VEGF) were purchased from Abcam (Cambridge, UK). Enhanced chemiluminescence (ECL) Streptavidin Horseradish Peroxidase conjugate was purchased from GE Healthcare (Little Chalfont, UK) and SuperSignal ELISA Pico Chemiluminescent Substrate was purchased from Pierce Biotechnology (Rockford, IL, USA).

Other reagents and solvents were purchased from commercial sources and used without further purification.

Deionized water was prepared in a Hydrolab water purification system (Hydrolab, Straszyn, Poland).

**Sc-44 radionuclide** was specially produced each time for this work and purified by us before each labeling process as described below. [^44^Sc]Sc was produced on a Ge-PETrace cyclotron (Heavy Ions Laboratory, Warsaw University, Warsaw, Poland) using 99.999% natural target CaCO_3_ in a ^44^Ca(p,n)^44^Sc irradiation reaction. The target material was pressed into a graphite support disc to increase heat conductivity and mechanical resistance. Irradiation was carried out for 2 h with 16 MeV protons with 14 µA current [25]. The separation of ^44^Sc from the target material was performed by two-step method based on microfiltration [42] and Dowex resin cation exchange [43] was used. First, the irradiated target was dissolved in 1 M HCl and then the target solution was alkalized with ammonia water (25%) to pH 11. In these conditions, scandium compounds were trapped on 0.22 µm porous PTFE (polytetrafluoroethylene membrane) microfilters. For the removal of calcium tracers, the microfilter was washed with 5 mL of deionized water. After that, ^44^Sc^III^ cations were removed from filters with 2 mL 1 M HCl and loaded on 200 mg of Dowex 50 wx4 resin. The bed was washed with 5 mL water for the removal of HCl. Following this, ^44^Sc^III^ was eluted with 0.4 M ready-to-use ammonium acetate buffer, pH 4.5 (in the form of [^44^Sc](CH_3_CHCOO)_3_Sc).

**Ga-68 radionuclide** in the form of [^68^Ga]GaCl_3_ in 0.1 M HCl was obtained from a ^68^Ge/^68^Ga generator (Eckert and Ziegler, Berlin, Germany) and was used right after the elution without additional concentration or purification steps.

**Lu-177 radionuclide** in the form of [^177^Lu]LuCl_3_ in 0.04 M HCl was purchased from National Centre for Nuclear Research Radioisotope Centre POLATOM (Świerk-Otwock, Poland) at a specific activity ≥ 370 GBq/mg Lu, in ready-to-use form.

### 2.2. Methods

#### 2.2.1. Analytical Methods

Conjugates **1** and **2** were analyzed by Knauer RP-HPLC in System 1 or 2, respectively, according to our previous work [41] and as stated below.

System 1: RP-HPLC analytical Eurospher-100-C-18 column (5 μm, 250 × 4.6 mm), solvent A: water with 0.1% trifluoroacetic acid (TFA, *v*/*v*), solvent B: acetonitrile/water (80:20, *v*/*v*) with 0.1% TFA (*v*/*v*), UV/Vis detection at 220 nm, gradient elution: 0–20 min 20 to 70% B, flow 1 mL/min.

System 2: RP-HPLC analytical Eurospher-100-C-18 column (5 μm, 250 × 4.6 mm), solvent A: water with 0.1% TFA (*v*/*v*), solvent B: acetonitrile/water (80:20, *v*/*v*) with 0.1% TFA (*v*/*v*), UV/Vis detection at 220 nm, gradient elution: 0–20 min 5 to 60% B, flow 1 mL/min.

Analyses of radioconjugates and their corresponding cold reference compounds were performed on a Shimadzu RP-HPLC using an analytical Phenomenex Jupiter 4u Proteo 90Å column (4 µm, 250 × 4.6 mm) or a semi-preparative Phenomenex Jupiter Proteo 90Å column (4 µm, 250 × 10 mm), and in both cases a Jupiter Proteo precolumn (20 × 2.1 mm), in System 3 for **^44^Sc-1**, **^44^Sc-1bis**, **^44^Sc-3**, **^68^Ga-3** and **^177^Lu-3** and System 4 for **^44^Sc-2** radioconjugate.

System 3: RP-HPLC semi-preparative Phenomenex Jupiter Proteo 90Å column (4 μm, 250 × 10 mm), gamma or UV/VIS detection (220 nm), solvent A: acetonitrile with 0.1% TFA (*v*/*v*), solvent B: water with 0.1% TFA (*v*/*v*), gradient elution: 0–20 min 20 to 80% A, 20–30 min 80% A, 30–32 min 80 to 20% A, flow 2 mL/min.

System 4: RP-HPLC analytical Phenomenex Jupiter 4u Proteo 90Å column (4 μm, 250 × 4.6 mm), gamma or UV/VIS detection (220 nm), solvent A: acetonitrile with 0.1% TFA (*v*/*v*), solvent B: water with 0.1% TFA (*v*/*v*), gradient elution: 0–20 min 1 to 50% A, 20–25 min 50 to 95% A, 25–31 min 95% A, 31–35 min 95 to 1% A, flow 1 mL/min.

#### 2.2.2. Syntheses

**Scheme 1:**
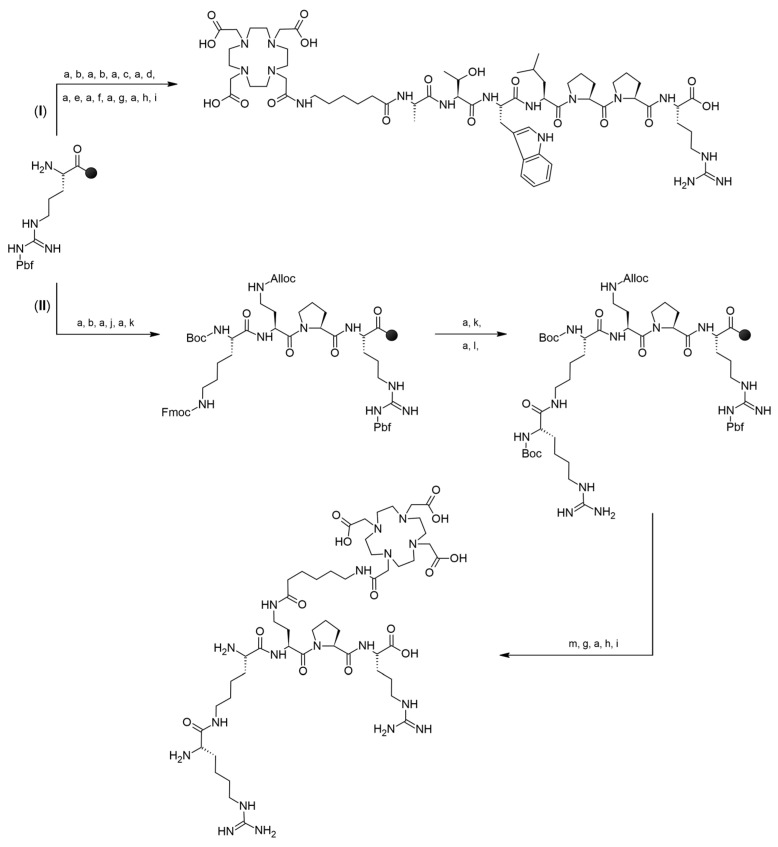
Synthesis strategy of conjugate **1** (path I) and **2** (path II) by SPPS method. (**a**)—30% piperidine in DMF, (**b**)—Fmoc-Pro-OH:DIC:HOBt, (**c**)—Fmoc-Leu-OH:DIC:HOBt, (**d**)—Fmoc-Trp(Boc)-OH:DIC:HOBt, (**e**)—Fmoc-Thr(tBu)-OH:DIC:HOBt, (**f**)—Fmoc-Ala-OH:DIC:HOBt, (**g**)—Fmoc-Ahx-OH:DIC:HOBt, (**h**)—DOTA-tris(tBu)-NHS:triethylamine, (**i**)—TFA/PhOH/H_2_O/TIPS, (**j**)—Fmoc-L-Dab(Alloc)-OH:DIC:HOBt, (**k**)—Boc-Lys(Fmoc)-OH:DIC:HOBt, (**l**)—di-Boc-S-methylisothiourea:n-butylamine:DCM, (**m**)—phenylsilane:tetrakis(triphenylphosphine)-palladium:DCM [41].

Peptide Ala-Thr-Trp-Leu-Pro-Pro-Arg (A7R) and peptidomimetic Lys(hArg)-Dab-Pro-Arg (K4R) as well as their conjugates with Ahx linker and DOTA chelator (DOTA-Ahx-A7R—conjugate **1**, and DOTA-Ahx-K4R—conjugate **2**) were synthesized manually by SPPS method on the preloaded Fmoc-Arg(Pbf)-Wang resin following the Fmoc chemistry with DIC/HOBt coupling method and active ester method for coupling DOTA-tris(tBu)-NHS [41]. In the case of conjugate **1** (DOTA-Ahx-A7R), a linear chain was built first, and then without cleavage peptide from the resin, the linker and chelator were attached to the N-terminus of the alanine residue (Path I in Scheme 1). Additionally, in the case of conjugate **2** (DOTA-Ahx-K4R containing branched K4R peptidomimetic) synthesis, the main linear chain was first built, and next it was extended by two branches: first at the N-terminus by adding lysine residue (as a Boc-Lys(Fmoc) building block), which was then guanylated after deprotection of the Fmoc group, and second in the 2,4-diaminobutyric acid (Dab) side chain, where Fmoc-Ahx-OH and DOTA-tris(tBu)-NHS ester were added (Path II in Scheme 1). All detailed information is thoroughly described in our previous publication [41]. Retaining the N-terminal fragment of Lys(hArg) unchanged was necessary to maintain high affinity binding to the NRP-1 co-receptor, according to data obtained in [38].

##### Preparation of [^44^Sc]Sc-DOTA-Ahx-A7R (**^44^Sc-1**), [^44^Sc]Lys(DOTA-Sc)-A7R (**^44^Sc-1bis**), [^44^Sc]Sc-DOTA-Ahx-K4R (**^44^Sc-2**) and [^44^Sc]^D^R7A-^D^Lys(DOTA-Sc) (**^44^Sc-3**)

All ^44^Sc-radioconjugates were synthesized according to the following procedure: into the vial containing 25 nmol of lyophilized conjugate (**1**, **1bis**, **2** or **3**) the 150–250 μL solution of [^44^Sc](CH_3_CHCOO)_3_Sc (10–20 MBq) was added. The reaction mixture at pH 4.5 was heated for 20 min at 95 °C. The reaction progress was checked by RP-HPLC in System 3 with gamma detection for **^44^Sc-1, ^44^Sc-1bis** and **^44^Sc-3** and in System 4 with gamma detection for **^44^Sc-2**.

##### Preparation of [^68^Ga]Lys(DOTA-Ga)-A7R (^68^Ga-1bis) and [^68^Ga]^D^R7A-^D^Lys(DOTA-Ga) (^68^Ga-3)

A quantity of **^68^Ga-1bis** and **^68^Ga-3** radioconjugates was synthesized according to the following procedure: into the vial containing 25 nmol of lyophilized conjugate (**1bis** or **3**), the 300 µL of 0.4 M acetate buffer solution (pH 4.5), and 250 µL of the [^68^Ga]GaCl_3_ (15–30 MBq) were added. The reaction mixture at pH 3.5–4.0 was heated for 10 min at 95 °C. The reaction progress was checked by RP-HPLC in System 3 with gamma detection.

##### Preparation of [^177^Lu]Lys(DOTA-Lu)-A7R (**^177^Lu-1bis**) and [^177^Lu]^D^R7A-^D^Lys(DOTA-Lu) (**^177^Lu-3**)

A quantity of **^177^Lu-1bis** and **^177^Lu-3** radioconjugates were synthesized according to the following procedure: into the vial containing 2 nmol of lyophilized conjugate (**1bis** or **3**), the 200 µL of 0.4 M acetate buffer solution (pH 4.5), and 1.5–15 µL of the [^177^Lu]LuCl_3_ (5–15 MBq) were added. The reaction mixture at pH 4.5 was heated for 10 min at 95 °C. The reaction progress was checked by RP-HPLC in System 3 with gamma detection.

##### Preparation of Cold Reference Compounds Sc-DOTA-Ahx-A7R (**Sc-1**), Lys(DOTA-Sc)-A7R (**Sc-1bis**), Lys(hArg)-Dab(Ahx-DOTA-Sc)-Pro-Arg (**Sc-2**), ^D^R7A-^D^Lys(DOTA-Sc) (**Sc-3**) and ^D^R7A-^D^Lys(DOTA-Ga) (**Ga-3**) for ELISA Tests

To obtain the cold compounds for ELISA tests as well as to verify the identity of the **^44^Sc-1**, **^44^Sc-1bis**, **^44^Sc-2**, **^44^Sc-3** and **^68^Ga-3** radioconjugates, the analogues with stable scandium and gallium isotopes under the same reaction conditions were synthesized and analyzed by the RP-HPLC method (System 3 or 4 with UV/Vis detection) and ESI-MS method.

The reference compounds Sc-DOTA-Ahx-A7R (**Sc-1**), Lys(DOTA-Sc)-A7R (**Sc-1bis**), Lys(hArg)-Dab(Ahx-DOTA-Sc)-Pro-Arg (**Sc-2**) and ^D^R7A-^D^Lys(DOTA-Sc) (**Sc-3**) were synthesized according to the following procedure: into a vial containing approximately 1 mg (740–860 nmol) of a given conjugate (DOTA-Ahx-A7R, Lys(DOTA)-A7R, DOTA-Ahx-K4R and ^D^R7A-^D^Lys(DOTA), respectively) dissolved in 300–400 µL of 0.4 M acetate buffer (pH 4.5), the 4-fold excess of 1 mg/mL ScCl_3_ solution in 0.1 M HCl was added. The reaction mixture at pH 4.5 was heated for 20 min at 95 °C. The reaction progress was checked by RP-HPLC in System 3 or 4.

Reference compound ^d^R7A-^d^Lys(DOTA-Ga) (**Ga-3**) was synthesized according to the following procedure: to a vial containing approximately 740 nmol of conjugate ^D^R7A-^D^Lys(DOTA) dissolved in 200 µL of 0.4 M acetate buffer (pH 4.5), the 4-fold excess of 1.34 mg/mL GaCl_3_ solution in 0.065 M HCl was added. The reaction mixture at pH 3.5–4.0 was heated for 10 min at 95 °C. The reaction progress was checked by RP-HPLC in System 3.

#### 2.2.3. Physicochemical Properties Study of the Radioconjugates

A necessary requirement for both basic laboratory research as well as the routine practice in nuclear medicine of the use of radiopharmaceutical preparations is the performance of appropriate tests in vitro to assess the safety, quality and effectiveness of the radiopharmaceuticals used. In accordance with requirements for potentially novel radiopharmaceuticals and preferred intravenous application, radioconjugates **^44^Sc-1**, **^44^Sc-1bis**, **^44^Sc-2**, **^44^Sc-3**, **^68^Ga-3**, **^177^Lu-3, ^68^Ga-1bis** and **^177^Lu-1bis** were isolated from the reaction mixture and tested for their lipophilicity, stability in solutions imitating human body fluids and in human serum. Due to the presence of a radioactive isotope in the radiocompound structure in nanomolar amounts, procedures for physicochemical properties testing of novel potential radiopharmaceuticals are carried out with the use of very small amounts (nmol) of radiocompounds. This creates additional difficulties in performing experiments (detection methods), and the results obtained are not always exactly the same as in the case of experiments conducted on a macro scale (mmol). All studies of the physicochemical properties of the obtained radiopreparations were carried out with their solutions in 0.1 M PBS (pH 7.4).

##### Radioconjugate Stability Tests

Radioconjugate stability tests in 0.1 M PBS solution, 1 mM cysteine (Cys) solution, 1 mM histidine (His) solution and human serum (HS) were performed under rigorous conditions according to the procedure described in detail in our previous paper [41]. In brief, isolated radioconjugates were dissolved in 50 µL PBS and appropriate volume of Cys, His and HS solution and then incubated at 37 °C. After the specified incubation time (depending on the half-life of a given radionuclide), which was up to 4–8 h for the ^44^Sc-radiocompounds, 4 h for the ^68^Ga-radiocompounds and about 6–14 days for the ^177^Lu-radiocompounds, aliquots of solution were withdrawn from the mixture and the composition of the solution was analyzed on RP-HPLC in System 3 or 4 with gamma detection. The concentrations of Cys and His solutions were about 1000 times higher than that of the studied radiocompound. In the case of the radioconjugate stability study in HS, the concentration of the radiocompound was a maximum of 2–25 nmol/mL in 100% human serum. After incubation, the protein components were precipitated with a 2.5-fold volume excess of ethanol prior to RP-HPLC analysis, and then both the supernatant and precipitate radioactivity were measured with a Wizard gamma counter. The percentage of radioactivity remaining in the supernatant and bound by the precipitated serum protein components was calculated as the ratio of the radioactivity of the supernatant or the precipitate, respectively, to the sum of the radioactivity of the supernatant and the precipitate.

##### Lipophilicity Test

The lipophilicity value, defined as the decimal logarithm (logP) of the partition coefficient (P), was tested in the system of two immiscible (but saturated with each other) phases: 0.1 M PBS solution, pH 7.4, and *n*-octanol, in three independent experiments. Partition coefficient was determined as a ratio of radioactivity of the organic (*n*-octanol) and aqueous (PBS solution) phases and was shown as the mean ± SD. The lipophilicity test was carried out as follows: the tested radiocompound dissolved in 10–20 µL of PBS was incubated and intensively mixed in an equal amount of these two phases. After centrifugation, radioactivity of appropriate amounts of the separated organic (500 µL) and aqueous (100 µL) phases were measured on the Wizard gamma counter. Differences in the volume of phases taken for measurement resulted from the very low radioactivity of organic phases and were included in the calculations.

Additionally, to confirm the stability of the studied radiocompounds during the experiment, the RP-HPLC analyses of the aqueous phase were performed after each lipophilicity test.

#### 2.2.4. Competitive NRP-1-Binding Inhibition Assay

The study of biological activity (inhibition of VEGF-A_165_/NRP-1 binding) of **Sc-1**, **Sc-1bis**, **Sc-2**, **Sc-3** and **Ga-3** compounds was tested by competitive ELISA test. The method used was similar to the one previously described [44,45]. Briefly, the first step was to coat the bottom surface of a 96-well plate using 100 µL (200 ng/well) of recombinant human NRP-1 and incubate overnight at 4 °C. Nonspecific binding was blocked with 0.5% BSA (bovine serum albumin) in PBS. Following this, 50 µL of the solution of tested compound in PBS, in the selected concentrations (100 [µM], 50 [µM], 25 [µM], 12.5 [µM] and 6.75 [µM]), and 50 µL (400 ng/mL) of human (bt)-VEGF-A_165_ in PBS containing 4 µg/mL of heparin were added. After 2 h of incubation at room temperature, the plate was washed and treated with a streptavidin–horseradish peroxidase conjugate in PBS (1:100) for 45 min. After the final wash, the chemiluminescence was quantified immediately after addition of 100 μL chemiluminescent substrate. Wells incubated with (bt)-VEGF-A_165_ served only as positive control (P), while wells not coated with NRP-1 were used as negative control (NS). Percentages of inhibition were calculated according to the following formula.

$$\%\;inhibition=100\%\;-\frac{\left(S-NS\right)\cdot 100\%}{P-NS}$$

where: *S*—measured signal, *NS*—negative control signal, *P*—positive control signal.

Test IC_50_ [µM] values (mean ± SD of three independent experiments) were determined by nonlinear regression analysis (log(inhibitor) vs. normalized response—variable slope) generated in GraphPad Prism 9.0.0 program (Graph Pad, San Diego, CA, USA).

## 3. Results

### 3.1. Syntheses of Conjugates

**Figure 1:**
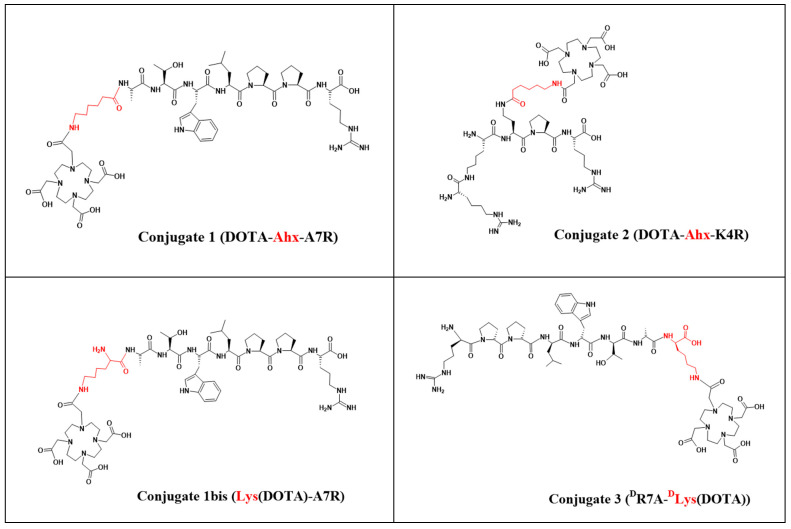
Chemical structure of the conjugates **1**, **1bis**, **2**, and **3**. The linkers Ahx and Lys are marked in red.

The syntheses of conjugates **1** (synthesis yield 37%, purity > 98%) and **2** (synthesis yield 27%, purity > 95%) are shown in Scheme 1 and were described in more detail in our previous work [41]. Conjugate **3** ((^D^R7A-^D^Lys(DOTA), based on ^D^R7A peptide—the retro-inverso isomer of the A7R peptide) was designed to be as structurally similar as possible to conjugate **1** (based on the A7R peptide). In this case, arginine (responsible for the effective interaction with the b1 subdomain of the NRP-1 binding pocket [14,18,31,32]) is located at the N-terminus of the ^D^R7A peptide, and the attachment of the chelator had to be via the C-terminal amino acid dAla. However, due to the fact that the ^D^R7A peptide in conjugate **3** has an inverted amino acid sequence, it was not possible to attach DOTA via the Ahx linker, as was in the case of conjugate **1** [41]. Therefore, it has been proposed to use the dLys amino acid as a linker, due to its structural similarity to Ahx and the possibility of attaching a chelator via the epsilon amino group of dLys (Figure 1). Additionally, in order to be able to assess the impact of replacing the Ahx linker with the Lys linker, a new conjugate **1bis** (Lys(DOTA)-A7R) was designed containing as a linker amino acid Lys instead of Ahx (Figure 1). All conjugates were purified by RP-HPLC (Table 1) and analyzed by ESI-MS.

**ESI-MS for conjugate 1**, *m/z*: ion found [M+3H]^3+^: 447.5, calculated [M+3H]^3+^: 447.0; found [M+2H]^2+^: 670.7, calculated [M+2H]^2+^: 670.0; found [M-2H]^2^: 668.6, calculated [M-2H]^2^: 668.0; found [M-H]: 1337.9, calculated [M-H]^−^: 1337.9.

**ESI-MS for conjugate 1bis**, *m/z*: ion found [M+H]^+^: 1355.8, calculated [M+H]^+^: 1355.6.

**ESI-MS for conjugate 2**, *m/z*: ion found [M+4H]^4+^: 293.2, calculated [M+4H]^4+^: 293.0; found [M+3H]^3+^: 390.6, calculated [M+3H]^3+^: 390.3; found [M+2H]^2+^: 585.4, calculated [M+2H]^2+^: 585.0; found [M-H]^−^: 1167.7, calculated [M-H]^−^: 1167.0.

**ESI-MS for conjugate 3**, *m/z*: ion found [M+H]^+^: 1355.2, calculated [M+H]^+^: 1355.6.

### 3.2. Syntheses of Radioconjugates

**Figure 2:**
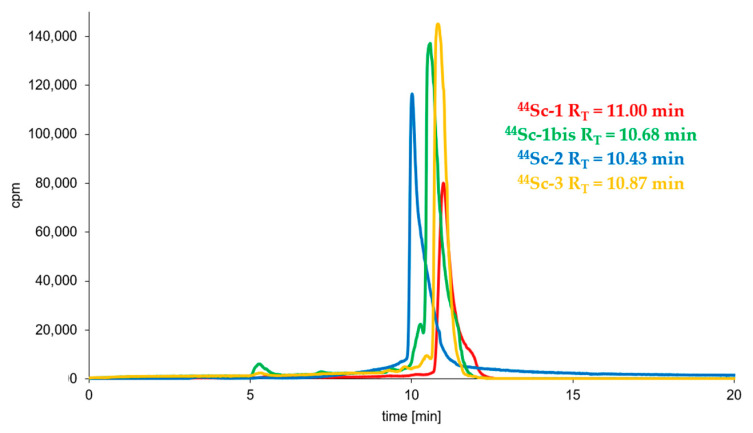
RP-HPLC radiochromatograms of the **^44^Sc-1**, **^44^Sc-1bis**, **^44^Sc-3** (System 3) and **^44^Sc-2** (System 4) radioconjugates.

Syntheses of all studied ^44^Sc-radioconjugates based on A7R, K4R and ^D^R7A biomolecules were performed (using previously freeze-dried ready-to-use conjugate kits) with the high radiochemical yield (>95%) and high radiochemical purity (>95%) and were analyzed by RP-HPLC in a dedicated System 3 or 4 (Table 1). Figure 2 shows the RP-HPLC radiochromatograms obtained for **^44^Sc-1**, **^44^Sc-1bis**, **^44^Sc-2**, and **^44^Sc-3** radioconjugates (the radiochromatograms of **^68^Ga-1**, **^68^Ga-2**, **^177^Lu-1** and **^177^Lu-2** radioconjugates have already been presented in our previous publication [41]). All radiochromatograms show only single peak, which confirms the correctness of the selected synthesis conditions with a quick and efficient high quality preparation procedure from previously prepared lyophilizates.

### 3.3. Radioconjugates Stability Studies in PBS Buffer, Cysteine, Histidine and Human Serum

The incubation of all radioconjugates in 0.1 M PBS, pH 7.4, solution showed that all synthesized radiocompounds are stable in PBS solutions (only one peak with retention time (R_T_) corresponding to the R_T_ of the tested radiocompound that was recorded in a radiochromatogram).

**Figure 3:**
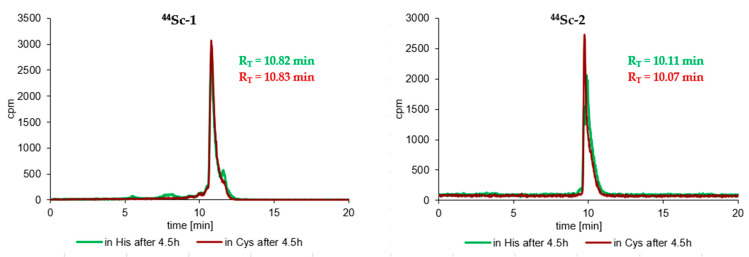

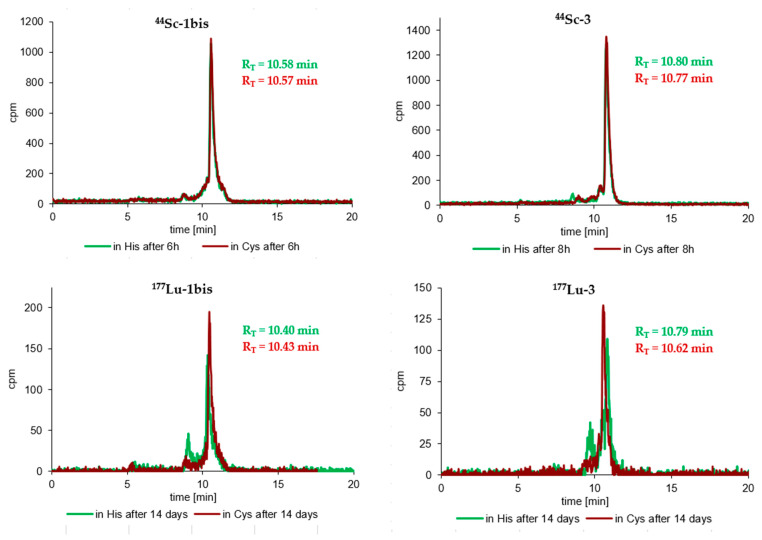
RP-HPLC radiochromatograms of cysteine and histidine stability tests of the **^44^Sc-1**, **^44^Sc-1bis**, **^44^Sc-3, ^177^Lu-1bis**, **^177^Lu-3** (System 3) and **^44^Sc-2** (System 4) radioconjugates after the longest incubation time for a given radioconjugate.

Similarly, very satisfactory results were also obtained in the stability tests of radioconjugates against ligand exchange reactions in Cys and His solutions. Practically all radioconjugates were completely stable in these two solutions up to an appropriate incubation time (up to 4–8 h for the ^44^Sc-radiocompounds, 4 h for the ^68^Ga-radiocompounds and about 6–14 days for the ^177^Lu-radiocompounds), and at the end of the incubation process only trace amounts of transchelatation products in His solution were observed in RP-HPLC radiochromatograms. The results of the stability study of the tested ^44^Sc- and ^177^Lu-radioconjugates in Cys and His solutions after maximal incubation times are presented in Figure 3.

**Figure 4:**
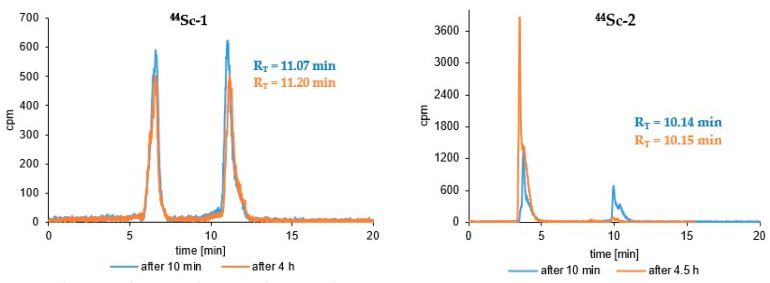

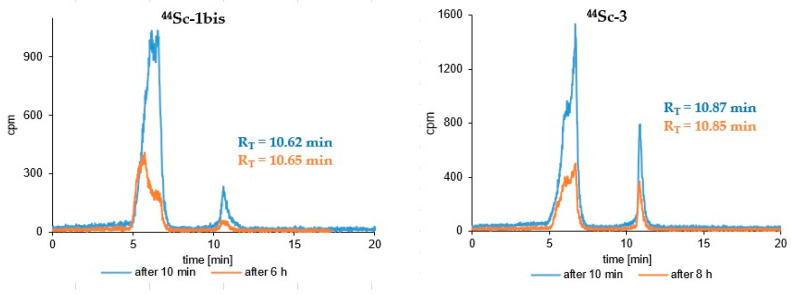
RP-HPLC radiochromatograms of human serum stability tests of the **^44^Sc-1**, **^44^Sc-1bis**, **^44^Sc-3** (System 3) and **^44^Sc-2** (System 4) radioconjugates after different incubation times.

**Figure 5:**
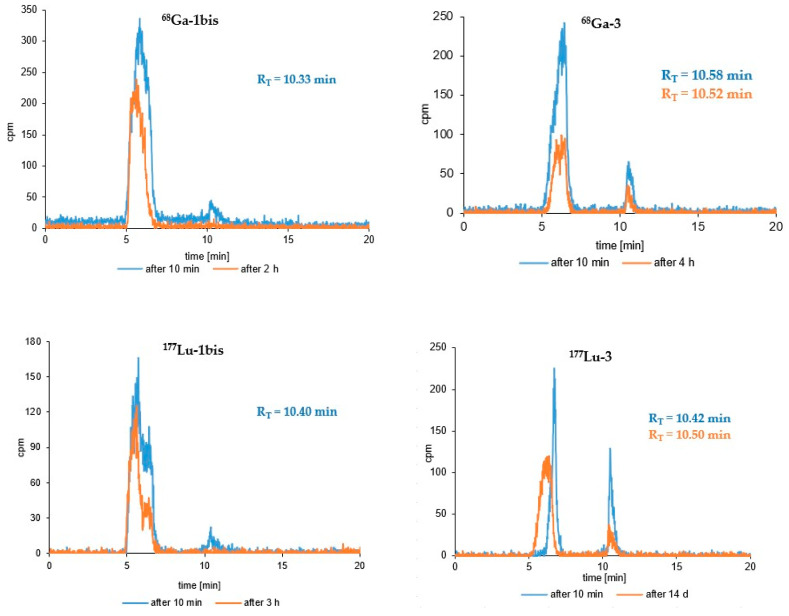
RP-HPLC radiochromatograms of human serum stability tests of the **^68^Ga-1bis**, **^177^Lu-1bis**, **^68^Ga-3** and **^177^Lu-3** (System 3) radioconjugates after different incubation times.

Results of the stability study of the tested radioconjugates in human serum after selected representative incubation times are presented in Figure 4 and Figure 5. Radiocompounds based on A7R and K4R biomolecules are exposed to generally low stability against proteolytic enzymes. Radiocompounds based on the A7R peptide show much greater proteolytic stability compared to those based on the peptidomimetic K4R (Figure 4 and Figure 5); however, in both cases their disintegration profile in this medium is too fast and unacceptable. Radioconjugates containing the ^D^R7A peptide as a biomolecule also show a relatively fast biodegradation profile, although definitely slower than in the case of radiocompounds based on A7R and K4R biomolecules (Figure 4 and Figure 5).

Graphical stability plots of **^68^Ga-1**, **^68^Ga-2**, **^177^Lu-1** and **^177^Lu-2** radioconjugates have already been presented in our previous publication [41].

### 3.4. Lipophilicity Study

**Figure 6:**
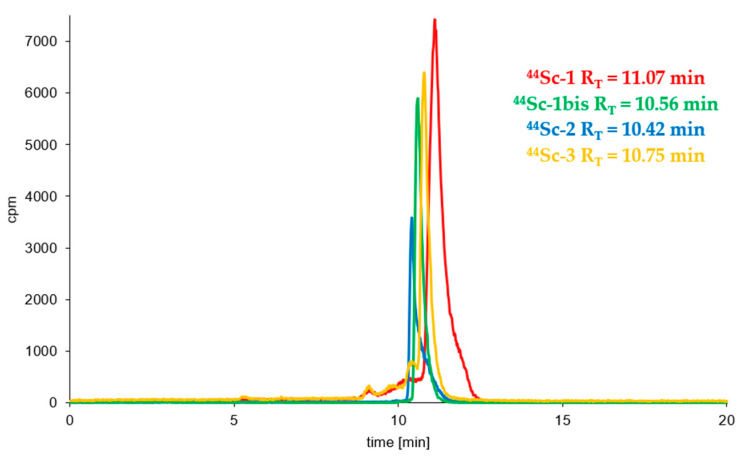
RP-HPLC radiochromatograms of the aqueous phase after lipophilicity experiment for **^44^Sc-1**, **^44^Sc-1bis**, **^44^Sc-3** (System 3) and **^44^Sc-2** (System 4) radioconjugates.

The lipophilicity values (logP) obtained for all tested radioconjugates are presented in Table 1. Generally, such significantly low values (logP in the range from −2.57 to −4.57) indicate a hydrophilic nature for all tested radiocompounds. The obtained radiochromatograms of RP-HPLC analyses of the aqueous phases for all tested radioconjugates, performed after the end of the lipophilicity test, showed only one peak, with R_T_ corresponding to the appropriate radiocompound, which confirms the radioconjugates’ stability under experiment conditions. Radiochromatograms of the aqueous phases for ^44^Sc-radiocompounds are presented in Figure 6.

### 3.5. Syntheses of Cold Reference Compounds

The results of ESI-MS analyses of cold reference compounds (synthesized under the same conditions as radiocompounds) are presented below. Comparison of the retention times of cold reference compounds with the retention times of radioconjugates (Table 1) confirms the obtaining of the expected radiocompounds in the radiolabeling reactions as well as biomolecule stability under labeling reaction conditions (10 min or 20 min at 95 °C).

**ESI-MS for Sc-1**, *m/z*: ion found [M+3H]^3+^: 461.2, calculated [M+3H]^3+^: 461.0; found [M+2H]^2+^: 691.3 calculated [M+2H]^2+^: 691.0.

**ESI-MS for Sc-1bis**, *m/z*: ion found [M+H]^+^: 1396.7, calculated [M+H]^+^: 1396.5.

**ESI-MS for Sc-2**, *m/z*: ion found [M+3H]^3+^: 404.6, calculated [M+3H]^3+^: 404.3; found [M+2H]^2+^: 606.3, calculated [M+2H]^2+^: 606.0.

**ESI-MS for Sc-3**, *m/z*: ion found [M+H]^+^: 1396.6, calculated [M+H]^+^: 1396.5.

**ESI-MS for Ga-3**, *m/z*: ion found [M+H]^+^: 1420.7, calculated [M+H]^+^: 1420.6; found [M+H]^+^: 1422.7, calculated [M+H]^+^: 1422.6.

**ESI-MS for Lu-3**, *m/z*: ion found [M+H]^+^: 1526.7, calculated [M+H]^+^: 1526.5.

### 3.6. Biological Activity Study with ELISA Competitive Test

The studies of the biological activity of ^44^Sc-radiocompounds (**^44^Sc-1**, **^44^Sc-1bis**, **^44^Sc-2**, **^44^Sc-3** and **^68^Ga-3**) as a study of their inhibition of the VEGF-A_165_/NRP-1 complex formation were performed using their reference compounds **Sc-1**, **Sc-1bis**, **Sc-2**, **Sc-3** and **Ga-3** and a competitive ELISA test. The obtained IC_50_ values for the tested compounds (biomolecules and reference compounds based on them for synthesized radioconjugates) are presented in Table 2.

## 4. Discussion

The investigated properties of the designed and synthesized radioconjugates will be discussed in terms of the usefulness of the obtained radiocompounds as diagnostic/therapeutic radiopharmaceuticals in anti-angiogenic cancer diagnosis and therapy. According to the requirements for radiopharmaceuticals, the obtained radiopreparations should be characterized by high stability in human body fluids and blood, adequate lipophilicity and high affinity for a specific molecular target, and in this respect their physicochemical and biological properties will be discussed below.

Stability studies of the obtained radioconjugates (**^44^Sc-1**, **^44^Sc-1bis**, **^44^Sc-2**, **^44^Sc-3**, **^68^Ga-3**, **^177^Lu-3**, **^68^Ga-1bis** and **^177^Lu-1bis**) showed complete stability of all tested radiocompounds in PBS buffer and satisfactory resistance against ligand exchange reactions in 1000 times higher concentrated cysteine and histidine solutions. RP-HPLC radiochromatograms recorded at the end of the incubation processes showed only single peaks with R_T_ values corresponding to the appropriate radioconjugate (Figure 3). Slight traces of transchelation products were only observed in the radiochromatograms recorded at the longest incubation times (over 1 day) in the His solution (radiochromatograms of **^177^Lu-1bis** and **^177^Lu-3** in Figure 3), probably due to the presence of an imidazole ring (being an effective chelator of metal cations) in the His structure. A similar and noticeably lower stability of **^68^Ga-1**, **^68^Ga-2**, **^177^Lu-1** and **^177^Lu-2** radioconjugates in His solutions was also observed in our previous studies [41]. The achieved stability of all tested radiocompounds in these media fully meets the requirements for radiopharmaceuticals.

Stability studies of the obtained radioconjugates (**^44^Sc-1**, **^44^Sc-1bis**, **^44^Sc-2**, **^44^Sc-3**, **^68^Ga-3**, **^177^Lu-3**, **^68^Ga-1bis** and **^177^Lu-1bis**) in human serum (HS) showed, unfortunately, insufficient stability of all tested radiocompounds in this medium (Figure 4 and Figure 5). The main reason for the instability of radiopreparations in HS is the biodegradation of biological vectors (A7R and ^D^R7A peptides, and K4R peptidomimetic) under the influence of proteolytic enzymes present in HS. The relatively better stability was demonstrated by radiocompounds containing conjugate **3** (based on the retro-inverso ^D^R7A peptide). According to the literature, the ^D^R7A peptide composed of D-amino acids should be resistant to the action of enzymes, and according to the reports of Ying et al. [39], Jiao et al. [40] and Su et al. [46], the retro-inverso ^D^R7A peptide shows excellent stability in 25% mouse serum in PBS (while A7R is rapidly degraded under such conditions) [39], in 10% human serum [40] as well as in fetal bovine serum [46], respectively. Our stability tests showed that after 8 h of incubation in natural human serum of **^44^Sc-3** radiocompound (corresponding to two times the half-life of the ^44^Sc radionuclide), only about 20.5% of the radiocompound remained in intact form. Comparing the radiochromatograms for short-term incubation (4–8 h) for **^44^Sc-1** and **^44^Sc-3** radioconjugates (Figure 4), it would seem that the biodegradation of radiopreparations based on the A7R peptide (**^44^Sc-1**) and the ^D^R7A peptide (**^44^Sc-3**) occurs in a similar way. However, the use of the Lu-177 radionuclide (with a much longer t_1/2_, allowing for the observation of the biodegradation progress for 14 days) showed that **^177^Lu-1** was practically completely degraded in HS after 4 days of incubation (only about 2.6% of the **^177^Lu-1** radiocompound remained in the intact form [41]), whereas in the case of the **^177^Lu-3** radioconjugate, the unchanged form of this radiocompound after 14 days of incubation in HS was still present in this solution and was approximately 11.5% (Figure 5).

At this point, it is worth noting the difference in the scale of experiments performed for radiocompounds (described in the presented paper) and biomolecules (peptides and peptidomimetics described by other working groups) which, in our opinion, is the main reason for the differences in the human serum stability results obtained for biomolecules and radioconjugates based on them. As we wrote earlier, the main reason for the instability of radiopreparations in HS is the biodegradation of biomolecules used as vectors under the influence of proteolytic enzymes present in HS. Predicting the results of research conducted on the nano-scale (e.g., radiocompound stability study in human serum—in this work, 2–25 nmol of the conjugate was used for labeling, which resulted a maximal radiocompound concentration of 2–25 nmol/mL) based on the results obtained in the micro-scale (e.g., peptide or peptidomimetic stability study in human serum) may lead to erroneous conclusions, because the environmental conditions are completely different. In the first case, the amount of the tested radiocompound (determined by gamma detection) is several orders of magnitude lower than the amount of enzymes present in human serum; therefore, the effect of the biodegradation process of the unstable radiocompound is observed almost immediately. In the second case, the amount of the tested biomolecule is many times greater than the amount of enzymes; therefore, the presence of the biomolecule (determined by UV/VIS detection) in the unchanged form may be recorded after a longer period of time, which may erroneously suggest its certain stability in human serum.

In summary, ^D^R7A peptide-based ^68^Ga- and ^44^Sc-radioconjugates, approximately 50% stable in human serum for 4–5 h, can be considered potential radiopharmaceuticals for PET diagnostics.

Following this, as can be seen on the radiochromatograms shown in Figure 4 (radioconjugate **^44^Sc-1bis**) and Figure 5 (radioconjugates **^68^Ga-1bis** and **^177^Lu-1bis**) radiocompounds based on conjugate **1bis** (containing linker Lys instead of Ahx) were much less resistant to serum enzymes compared to those based on conjugate **1**. After 4 h of **^44^Sc-1** radioconjugate incubation in HS, only about 50% of the radiocompound was decomposed, while in the case of **^44^Sc-1bis** this value increased significantly to almost 92% in the same incubation time. Similar results were also obtained for ^68^Ga-radioconjugates—about 63% of **^68^Ga-1** was decomposed after 4.5 h of incubation in HS [41], while **^68^Ga-1bis** was completely degradable by serum enzymes after just 2 h incubation (Figure 5). In the case of ^177^Lu-radioconjugates, **^177^Lu-1** almost completely disappeared on the RP-HPLC radiochromatograms after 4 day of incubation [41], while in the case of **^177^Lu-1bis**, the peak disappeared after 3 h (Figure 5). It therefore seems that the replacement of Ahx with Lys amino acid significantly increases the enzymatic biodegradation of the radioconjugate.

For all A7R and ^D^R7A peptide-based ^44^Sc-radioconjugates, the amounts of radioconjugates bound to serum protein components were similar—approximately 15–20%. Only in the case of radioconjugates based on the peptidomimetic K4R were these amounts higher (20–30%), which resulted from differences in the polarity of these biomolecules and was already discussed in detail in our previous work [41].

The lipophilicity values (logP) presented in Table 1 show that all tested radioconjugates are clearly hydrophilic in nature. The lipophilicity of a molecule is not an additive quantity derived from individual fragments of the structure of this molecule; nevertheless, each of these fragments contributes to the resultant logP value. It is worth noting that radioconjugates containing the amino acid Lys as a linker in their structure (**^68^Ga/^177^Lu/^44^Sc-1bis** radioconjugates based on conjugate **1bis**) in comparison to radioconjugates containing as a linker the Ahx molecule (**^68^Ga/^177^Lu/^44^Sc-1** radioconjugates based on conjugate **1**) are characterized with lower logP values. It can be concluded that replacing the linker (from Ahx to Lys) increases the hydrophilicity and consequently reduces lipophilicity values of the tested radiocompounds. The same effect of the presence of Lys on the compound lipophilicity can be observed when analyzing the logP values of radioconjugates **^68^Ga/^177^Lu/^44^Sc-1bis** (containing conjugate **1bis**) and **^68^Ga/^177^Lu/^44^Sc-3** (containing conjugate **3**) in comparison to that of radioconjugates **^68^Ga/^177^Lu/^44^Sc-1** (containing conjugate **1**) and **^68^Ga/^177^Lu/^44^Sc-2** (containing conjugate **2**). Statistically logP values of radioconjugates containing conjugates **1bis** and **3** (with Lys amino acid as a linker) are lower than logP values of radioconjugates containing conjugates **1** and **2** (with Ahx molecule as a linker).

The lower logP values of radioconjugates containing **1bis** and **3** conjugates in their structure in comparison to those of radioconjugates containing conjugate **1** may be also a result of the presence of an additional free amine groups in the structures of conjugates **1bis** and **3** (α-amine group of Lys linker in conjugate **1bis** and α-amine group of N-terminal DArg in conjugate **3**).

Comparing the lipophilicity of the radioconjugates in terms of the radionuclide used, it can be seen that, in general, radioconjugates containing the cations ^177^Lu^III^ or ^44^Sc^III^ (both with coordination number of 8) turned out to be more lipophilic than radioconjugates containing the ^68^Ga radionuclide. Furthermore, ^68^Ga isotope-containing radioconjugates are characterized in general with the lowest lipophilicities, probably due to the presence of the free COOH group (of DOTA chelator) not participating in the complexation of the ^68^Ga^III^ cation (with a coordination number of 6), which results in an increase in the hydrophilicity of the radioconjugate molecules.

Concluding the discussion on the lipophilicity of the studied radioconjugates, it could be noted that all tested radiocompounds are characterized by a low lipophilicity (logP values are in the range from −2.57 to −4.57, Table 1), which generally does not meet the requirements for radiopharmaceuticals dedicated to targeting to the surface receptors. Radiopreparations with such low lipophilicity will generally display poor ADME (administration, distribution, metabolism, excretion) properties and would not be able to cross the blood–tissue and blood–brain barrier which is necessary for the distribution of the radiocompound to such receptors. However, as we explained in our previous work [41], this inability to cross the blood–tissue barrier is not a crucial parameter in this case, because VEGF-A_165_ is present in the endothelium, the single layer of squamous endothelial cells that lines the interior surface of blood vessels; thus, it can directly interact with components (e.g., administered radiopharmaceuticals) present in the blood. In addition, in this case, the low lipophilicity parameter of the tested radiocompounds is to their advantage, as it positively affects the elimination of unbound radiopharmaceuticals, which increases their safety profile, especially in the case of radiocompounds containing long-lived therapeutic radionuclides in their structure (e.g., ^177^Lu with t_1/2_ 6.65 d).

Due to the chemical identity of **^44^Sc-1**, **^44^Sc-1bis**, **^44^Sc-2**, **^44^Sc-3** and **^68^Ga-3** radioconjugates and their reference compounds **Sc-1**, **Sc-1bis, Sc-2**, **Sc-3** and **Ga-3** (differing only in the metal isotope used), the study of VEGF-A_165_/NRP-1 complex formation inhibition by **^44^Sc-1**, **^44^Sc-1bis**, **^44^Sc-2**, **^44^Sc-3** and **^68^Ga-3** radioconjugates could be performed using their reference compounds. Inhibition studies of VEGF-A_165_ binding to NRP-1 performed using the competitive ELISA test were carried out for two series of compounds, i.e., the parent peptides A7R, ^D^R7A and parent branched peptidomimetic K4R, and the compounds **Sc-1**, **Sc-1bis**, **Sc-2**, **Sc-3** and **Ga-3** listed above.

The previous study performed by our research group indicated that the K4R peptidomimetic shows high affinity to NRP-1 due to the introduction of the hArg via the side chain (ε amine group) of Lys at the N-terminus and the unnatural Dab at the second position, which together result in the adoption of the optimal conformation of K4R molecule for binding to NRP-1 [38]. According to the literature, the retro-inverso ^D^R7A peptide shows similar binding affinity for VEGFR-2 and NRP-1 compared to its parent peptide A7R [39,40].

The results of our studies of inhibition of the VEGF-A_165_/NRP-1 complex formation for the tested compounds (Table 2) show high binding affinity for NRP-1 in the case of A7R, K4R, **Sc-1**, **Sc-1bis** and **Sc-2** compounds and a very low binding affinity in the case of the compounds ^D^R7A, **Sc-3** and **Ga-3**. The IC_50_ values of **Sc-1**, **Sc-1bis** and **Sc-2** are slightly (and acceptably) higher than those of the parent compounds A7R and K4R, respectively. This allows us to conclude that despite the modification, the biologically active molecules A7R and K4R contained in the **Sc-1**, **Sc-1bis** and **Sc-2** compounds retain their biological properties, and therefore these radioconjugates are equally good inhibitors of the VEGF-A_165_/NRP-1 complex formation. The results obtained for **Ga-3** and **Sc-3** compounds (based on retro-inverso ^D^R7A peptide) clearly indicate that their property of inhibiting the VEGF-A_165_/NRP-1 complex formation is distinctly lower and this results from the low affinity of the parent ^D^R7A biomolecule. This certain very low affinity of the ^D^R7A peptide to NRP-1, as well as the radioconjugates based on it, is probably due to the lack of affinity of these derivatives for the b1 subdomain of the NRP-1 binding pocket. The ^D^R7A peptide is a retro-inverso analog of the A7R peptide, and the C-terminal arginine (with a free carboxyl group in A7R) needed to interact with NRP-1, now located at the N-terminus of the ^D^R7A peptide, has the D configuration and free amine group instead of carboxyl group. As a result of this spatial arrangement (D configuration, lack of COOH) it probably does not fit into the typical binding pocket in NRP-1 for A7R. Unfortunately, our results of studies on the affinity of the retro-inverso isomer of the ^L^A7R peptide and radioconjugates based on it for the NRP-1 co-receptor are not fully consistent with the literature [39,40], but they are consistent with the rule that for interaction with the binding pocket of NRP-1 the presence of terminal arginine with a free carboxyl group is necessary [14,18,31,32,33].

Based on the data obtained for **Sc-1**, **Sc-1bis** and **Sc-2** (Table 2), it can also be concluded that the addition of a linker (Ahx or Lys) and a metal (^68^Ga, ^177^Lu, ^44^Sc) complex to the parent peptide (A7R) or peptidomimetic (K4R) only slightly lowers the inhibitory properties of the parent compound. Comparing the IC_50_ values for **Sc-1** and **Sc-1bis**, it is also worth noting that, unlike the effect of replacing the Ahx linker with Lys on the parameter of radioconjugate stability in HS (Figure 4 and Figure 5) and radioconjugate lipophilicity (Table 1), in the case of the inhibitory activity of the tested compounds for the formation of the VEGF/NRP-1 complex, the linker replacement has no negative effect and compounds retain their high binding affinity corresponding to the affinity of the parent biomolecule.

## 5. Conclusions

All radioconjugates synthesized and tested in this study were obtained with high yield and radiochemical purity and found to be stable in Cys and His solutions mimicking human body fluids. Unfortunately, all radioconjugates proved to be insufficiently stable in human serum. Among the examined radiocompounds, only the **^68^Ga-1**, **^44^Sc-1**, **^68^Ga-3**, **^177^Lu-3** and **^44^Sc-3** radioconjugates, based on the A7R and retro-inverso ^D^R7A peptides, showed a certain stability. However, contrary to the literature on retro-inverso peptide [39,40,46], our results showed that this stability was low and unacceptable from the point of view of using these radioconjugates as receptor radiopharmaceuticals, especially in the case of therapeutic receptor radiopharmaceuticals.

Relatively similar and low IC_50_ values determined for the parent compounds A7R and K4R and radioconjugates based on them (**^44^Sc-1**, **^44^Sc-1bis** and **^44^Sc-2**) show that the resulting radioconjugates are good potential inhibitors of VEGF-A_165_/NRP-1 complex formation. In this respect, these radioconjugates could be used in cancer diagnosis and anti-angiogenic therapy; however, in the case of anti-angiogenic therapy, they are disqualified due to insufficient stability in human serum. On the other hand, the radioconjugates with a slightly improved HS stability (based on retro-inverso peptide ^D^R7A) practically (and contrary to the information in the literature), do not show the required affinity to NRP-1.

Considering the pivotal physicochemical (lipophilicity, stability in cysteine, histidine, human serum) and biological (affinity to NRP-1) properties determined for the radiopreparations tested in this study, it is worth noting that the properties of A7R peptide-based radioconjugates are most compliant with the requirements for diagnostic receptor radiopharmaceuticals, and if the enzymatic biodegradation profile in human serum of these radiopreparations were slower, their use in the diagnosis of pathological angiogenesis could be considered.

Our work on the development of new radiolabeled inhibitors of the VEGF-A_165_/NRP-1 complex formation is in progress; however, based on previous research, we suggest that non-peptide inhibitors of this complex formation should be sought.

## Figures and Tables

**Table 1:** R_T_ and logP (presented as the mean ± SD, *n* = 3) parameters determined for (radio)compounds tested in the present work. * radioconjugates synthesized and tested in [41].

(Radio)compound	RP-HPLC System	R_T_ [min]	log*P* ± SD
**^44^Sc-1**	3	11.00	−3.44 ± 0.02
**^44^Sc-1bis**	3	10.68	−3.56 ± 0.06
**^44^Sc-2**	4	10.43	−2.57 ± 0.02
**^44^Sc-3**	3	10.87	−3.61 ± 0.03
**^68^Ga-3**	3	10.72	−4.11 ± 0.06
**^177^Lu-3**	3	10.28	−4.48 ± 0.05
**^68^Ga-1bis**	3	10.32	−4.18 ± 0.07
**^177^Lu-1bis**	3	10.38	−4.45 ± 0.03
^68^Ga-1 *	3	11.27	−3.92 ± 0.03
^177^Lu-1 *	3	11.08	−3.40 ± 0.14
^68^Ga-2 *	4	10.63	−4.57 ± 0.05
^177^Lu-2 *	4	10.78	−3.75 ± 0.08
**Sc-1**	3	10.71	-----
**Sc-1bis**	3	10.26	-----
**Sc-2**	4	10.46	-----
**Sc-3**	3	10.60	-----
**Ga-3**	3	10.45	-----

**Table 2:** Inhibitory effects of **Sc-1**, **Sc-1bis**, **Sc-2**, **Sc-3** and **Ga-3** compounds on the VEGF-A_165_/NRP-1 complex formation in competitive ELISA test (presented as the mean ± SD, *n* = 3).

Biomolecule	Sequence	IC_50_ ± SD	logIC_50_ ± SD
or Reference Compound		[µM]	
**A7R**	Ala-Thr-Trp-Leu-Pro-Pro-Arg	17.47 ± 1.49	−4.74 ± 0.02
**Sc-1**	Sc-DOTA-Ahx-A7R	22.48 ± 6.56	−4.65 ± 0.12
**Sc-1bis**	Lys(DOTA-Sc)-A7R	18.08 ± 0.46	−4.74 ± 0.01
**K4R**	Lys(hArg)-Dab-Pro-Arg	4.83 ± 0.25	−5.32 ± 0.12
**Sc-2**	Sc-DOTA-Ahx-K4R	17.93 ± 1.80	−4.73 ± 0.03
**^D^R7A**	dArg-dPro-dPro-dLeu-dTrp-dThr-dAla	>100	n/a
**Ga-3**	^D^R7A-^D^Lys(DOTA-Ga)	>100	n/a
**Sc-3**	^D^R7A-^D^Lys(DOTA-Sc)	>100	n/a

## Data Availability

Not applicable.

## Abbreviations

A7R	Ala-Thr-Trp-Leu-Pro-Pro-Arg peptide
AAT	Anti-Angiogenic Therapy
ADME	administration, distribution, metabolism, excretion
Ahx	6-aminohexanoic acid
Alloc	Allyloxycarbonyl group
Boc	Tert-butyloxycarbonyl group
BSA	Bovine Serum Albumin
CendR	C-End-Rule
Cys	Cysteine
^D^R7A	DArg-DPro-DPro-DLeu-DTrp-DThr-DAla peptide
Dab	2,4-Diaminobutyric Acid
DCM	Dichloromethane
DIC	N,N’-Di(propan-2-yl)methanediimine
DMF	N,N-Dimethylformamide
DOTA	2,2′,2″,2″-(1,4,7,10-Tetraazacyclododecane-1,4,7,10-tetrayl) tetraacetic acid
DOTA-tris(tBu)-NHS	tri-tert-butyl 2,2′,2″-(10-(2-((2,5-dioxopyrrolidin-1-yl)oxy)-2-oxoethyl)-1,4,7,10-tetraazacyclododecane-1,4,7-triyl)triacetate
ECL	Enhanced chemiluminescence
ELISA	Enzyme-Linked Immunosorbent Assay
ESI-MS	Electrospray ionization mass spectrometry analyses
Et_3_N	Triethylamine
Fmoc	9-fluorenylmethoxycarbonyl group
hArg	Homoarginine
HCl	Hydrogen chloride
His	Histidine
HOBt	1H-1,2,3-Benzotriazol-1-ol
HS	Human serum
IC_50_	Half maximal inhibitory concentration
K4R	Lys(hArg)-Dab-Pro-Arg peptidomimetic
logP	Decimal logarithm of partition coefficient
m/z	Mass-to-charge ratio
NRP-1	Neuropilin-1
NS	Negative control
P	Partition coefficient
P	Positive control
Pbf	Pentamethyl-2,3-dihydrobenzofuran-5-sulfonyl group
PBS	Phosphate-buffered saline
PET	Positron Emission Tomography
PhOH	Phenol
PTFE	Polytetrafluoroethylene membrane microfilter
R/KXXR/K	Amino acid sequence containing: R-Arginine, K-Lysine, X- any amino acid
RP-HPLC	Reverse-Phase High Pressure Liquid Chromatography
R_T_	Retention time
S	Measured signal
SD	Standard Deviation
SPPS	Solid Phase Peptide Synthesis
t_1/2_	Radionuclide half-life time
tBu	Tert-butyl group
TFA	Trifluoroacetic acid
TIPS	Tri(propan-2-yl)silane
TRNT	Targeted Radionuclide Therapy
VEGF	Vascular Endothelial Growth Factor
VEGF-A_165_	Vascular Endothelial Growth Factor-A_165_
VEGFR-2	Vascular Endothelial Growth Factor Receptor 2
β^+^ E_max_	Beta emitter with a maximum energy
(bt)-VEGF-A_165_	Biotinylated VEGF-A_165_
(p,n)	Proton-neutron nuclear reaction
